# The utility of multivariate outlier detection techniques for data quality evaluation in large studies: an application within the ONDRI project

**DOI:** 10.1186/s12874-019-0737-5

**Published:** 2019-05-15

**Authors:** Kelly M. Sunderland, Derek Beaton, Julia Fraser, Donna Kwan, Paula M. McLaughlin, Manuel Montero-Odasso, Alicia J. Peltsch, Frederico Pieruccini-Faria, Demetrios J. Sahlas, Richard H. Swartz, ONDRI Investigators, Robert Bartha, Sandra E. Black, Michael Borrie, Dale Corbett, Elizabeth Finger, Morris Freedman, Barry Greenberg, David A. Grimes, Robert A. Hegele, Chris Hudson, Anthony E. Lang, Mario Masellis, William E. McIlroy, David G. Munoz, Douglas P. Munoz, J. B. Orange, Michael J. Strong, Sean Symons, Maria Carmela Tartaglia, Angela Troyer, Lorne Zinman, Stephen C. Strother, Malcolm A. Binns

**Affiliations:** 1 Rotman Research Institute, Baycrest Health Sciences, 3560 Bathurst St, Toronto, Ontario M6A 2E1 Canada; 2 Department of Kinesiology, University of Waterloo, 200 University Ave W, Waterloo, Ontario N2L 3G1 Canada; 3 Schulich School of Medicine and Dentistry, University of Western Ontario, 1151 Richmond St, London, Ontario N6A 5C1 Canada; 4 Gait and Brain Lab, Parkwood Institute, 550 Wellington Rd, London, Ontario N6C 0A7 Canada; 5 Lawson Health Research Institute, 750 Base Line Rd E, London, Ontario N6C 2R5 Canada; 6 Department of Medicine, McMaster University, 1280 Main St W, Hamilton, Ontario L8S 4L8 Canada; 7 Department of Medicine (Neurology), Sunnybrook Health Sciences Centre, 2075 Bayview Ave, Toronto, Ontario M4N 3M5 Canada; 8 Faculty of Medicine, University of Toronto, 1 King’s College Cir, Toronto, Ontario M5S 1A8 Canada; 9 Medical Biophysics Department, University of Toronto, 101 College St, Suite 15-701, Toronto, Ontario M5G 1L7 Canada; 10 Dalla Lana School of Public Health, University of Toronto, 155 College St, Toronto, Ontario M5T 3M7 Canada

**Keywords:** Quality control, Multivariate outliers, Minimum covariance determinant, Principal component analysis, Visualization

## Abstract

**Background:**

Large and complex studies are now routine, and quality assurance and quality control (QC) procedures ensure reliable results and conclusions. Standard procedures may comprise manual verification and double entry, but these labour-intensive methods often leave errors undetected. Outlier detection uses a data-driven approach to identify patterns exhibited by the majority of the data and highlights data points that deviate from these patterns. Univariate methods consider each variable independently, so observations that appear odd only when two or more variables are considered simultaneously remain undetected. We propose a data quality evaluation process that emphasizes the use of multivariate outlier detection for identifying errors, and show that univariate approaches alone are insufficient. Further, we establish an iterative process that uses multiple multivariate approaches, communication between teams, and visualization for other large-scale projects to follow.

**Methods:**

We illustrate this process with preliminary neuropsychology and gait data for the vascular cognitive impairment cohort from the Ontario Neurodegenerative Disease Research Initiative, a multi-cohort observational study that aims to characterize biomarkers within and between five neurodegenerative diseases. Each dataset was evaluated four times: with and without covariate adjustment using two validated multivariate methods – Minimum Covariance Determinant (MCD) and Candès’ Robust Principal Component Analysis (RPCA) – and results were assessed in relation to two univariate methods. Outlying participants identified by multiple multivariate analyses were compiled and communicated to the data teams for verification.

**Results:**

Of 161 and 148 participants in the neuropsychology and gait data, 44 and 43 were flagged by one or both multivariate methods and errors were identified for 8 and 5 participants, respectively. MCD identified all participants with errors, while RPCA identified 6/8 and 3/5 for the neuropsychology and gait data, respectively. Both outperformed univariate approaches. Adjusting for covariates had a minor effect on the participants identified as outliers, though did affect error detection.

**Conclusions:**

Manual QC procedures are insufficient for large studies as many errors remain undetected. In these data, the MCD outperforms the RPCA for identifying errors, and both are more successful than univariate approaches. Therefore, data-driven multivariate outlier techniques are essential tools for QC as data become more complex.

**Electronic supplementary material:**

The online version of this article (10.1186/s12874-019-0737-5) contains supplementary material, which is available to authorized users.

## Background

As technologies advance, the collection, management, curation, and analysis of large data become increasingly feasible, and large and complex studies are more prevalent as a result. In order for analyses to generate reliable results and allow for trustworthy conclusions, it is essential that these data be of high quality. For that reason, large-scale studies often include comprehensive quality assurance (QA) protocols to minimize occurrence of errors during data collection and preservation, as well as thorough quality control (QC) protocols to ensure accuracy once data have been collected.

Previous papers have discussed the importance of quality control [1–3], though details of the process are often minimal and success rates are rarely addressed. When details are given, they are often ad hoc and domain-specific [4], as opposed to a systematic process that can be applied to a variety of data types.

Numerous large and complex studies have been initiated in recent years, including the Canadian Longitudinal Study on Aging (CLSA) [5], the Alzheimer’s Disease Neuroimaging Initiative (ADNI) [6], and the Parkinson’s Progression Markers Initiative (PPMI) [7]. All three of these studies are multi-site initiatives with multiple types of data (including cognitive, neuroimaging, and genomics) and have the intent to share collected data openly with other researchers. While data sharing can stimulate the development of testable hypotheses or research designs [8] and improve reproducibility [4], it also substantially restricts the ability to verify data with source documents or assessment administrators when errors are suspected.

All three studies have internal statistical groups [9–11] that monitor data during acquisition and release stages, and generally report checking for anomalies, missing data, invalid entries, and general errors. While it is noted that QA and QC processes were undertaken [5, 12], there are not detailed descriptions of how they were performed, such as which techniques and approaches were used, or how these were applied to various types of data. Such information is important for consumers of these data, and would be useful to other groups collecting similarly large and complex multi-modal data.

Another such large and complex study is the Ontario Neurodegenerative Disease Research Initiative (ONDRI), a longitudinal, multi-site, and multi-cohort observational study that aims to characterize biomarkers within and between five neurodegenerative diseases [13]. The levels of disease, dysfunction, and decline are assessed for each participant across multiple measurement platforms (see Data below). The ONDRI baseline data have been acquired and we report on a data quality evaluation process based on multivariate outlier detection methods that was designed and used within ONDRI to ensure that data are of the highest possible accuracy. We also examine the sensitivity of univariate outlier detection to errors identified by the multivariate detection so that it may guide future large scale projects.

### Motivation for outlier detection as data quality evaluation

Data collection protocols usually include but are not limited to recording the measurement, transcribing components of the measurement, entering values into a database, calculating derived measures (e.g., standardized scores, summary scores), and subsequently distributing data. Many of these steps are performed manually and mistakes could feasibly be made during any stage, introducing erroneous values into the dataset [1]. When these steps are performed automatically human error is less likely, but errors can occur when equipment fails or through preprocessing steps, during which unstructured data may be misinterpreted (e.g., voice recordings). Herein, we consider any recorded value that does not accurately reflect the performance of the observed participant to be an error.

Common practices for ensuring data are accurate include manually checking the data for errors and double entry, where data are entered into the database twice by two different people and subsequently compared for accuracy. However, studies of the effectiveness of these techniques suggest that while they are successful in identifying some errors, they are often not sufficient as many errors remain [14, 15]. Also, manual procedures become increasingly time consuming as data grow in number of observations, number of variables, and/or variety of data types.

Data-driven processes that search for empirical relationships complement these typical data checking approaches. An error can often occur randomly (e.g., transcription errors) and an observation that possesses an error may appear distinct in relation to the other observations as a result. In such a case, the observation would qualify as an outlier under some statistical criteria. Outlier detection protocols use statistical techniques that identify patterns exhibited by the bulk of the data, and any data point that deviates from such a pattern becomes more evident. It could be a single value within a participant’s vector of data (e.g., a value that is several standard deviations away from the mean on a single variable), or as a result of the pattern exhibited by the relationships between multiple variables (e.g., a participant with values that depart from the covariance structure for the rest of the observations). To our knowledge, only one other paper recommends outlier detection for the purpose of error identification, but it does not provide methodological detail, nor the efficacy of the approach [16]. Outlier detection-based processes come with the caveat that they will not identify errors that do not appear distinct in relation to the other observations (i.e. are erroneous but appear typical). Further, correcting only errors that lie outside the general distribution reinforces the data relationships observed, without considering erroneous values within the bulk of the data that would be more accurately represented outside the distribution.

### Techniques for outlier detection

Some of the most common techniques for outlier detection focus on each variable independently, identifying extreme observations based on the observed univariate distribution. For example, when the distribution is assumed normal, observations are often considered outlying when they are beyond a pre-specified number of standard deviations from the mean [17, 18]. Since outliers can influence the estimated mean and standard deviation, their effect on these parameters could mask the degree to which they are outlying and they consequently may not be identified as outliers [19]. However, using robust methods for outlier detection can reduce or remove the effect of outliers on estimates of location and spread [17, 20]. One approach is truncation, where a pre-specified number of data points are excluded at both extremes of the distribution when calculating parameter estimates [21], consequently excluding any potential outliers and instead capturing the distribution of the majority of the data. Alternatively, methods based on the median, such as the Median Absolute Deviation or boxplots can be used as the extremity of an outlier is irrelevant to these robust measures of location and scale [19, 22]. However, because these approaches only consider each variable independently, they will not identify observations that are outlying given relationships between two or more variables. As a result, multivariate outlier detection methods may be valuable in most data sets.

Hadi, Rahtmatullah Imon, & Werner [23] suggest that multivariate outlier detection techniques fall into two general categories: methods based on distances and methods based on lower dimensional projections. One of the commonly used distance metrics in the multivariate space is the Mahalanobis Distance (MD), which considers the mean and covariance of the data, and for which larger distances are returned for observations that deviate from the mean in directions with smaller covariance [24]. However the influence of outliers is an issue with multivariate methods as well [25], so there exist a number of methods that first identify the robust distribution. These methods include the Minimum Volume Ellipsoid [26, 27] and the Minimum Covariance Determinant (MCD) [26, 27], where the MCD is more common and exhibits greater statistical efficiency and a faster convergence rate [28].

The goal of dimension-reduction based techniques is to extract “interesting” structure from a multivariate dataset by reducing the data to only a few informative dimensions [23]. The most well-known dimension-based technique is Principal Component Analysis (PCA) [29]. PCA has become a common tool for outlier detection as observations that do not fit the structure are exaggerated when projecting the data back onto the components. Further, it can handle data with more variables than observations, making it applicable to a more general selection of datasets. While PCA can be susceptible to influential values, many robust variations that are less susceptible to these effects have been introduced in recent years. These techniques often use regularization in some form to mitigate the effects of outliers and provide robust estimates of covariance. Some of these include regularized PCA [30], principal orthogonal complement thresholding [31], and Candès’ robust PCA (RPCA) [32].

For our data quality evaluation process we use two multivariate methods, one from each of the two general categories, and compare their selection of outliers and ensuing success in identifying errors with one another. We consider the MCD, coupled with Monte Carlo simulation and a corr-max transformation [33], and the RPCA, coupled with Orthogonal Distance measurements (see Methods). We do not propose that these are the optimal methods, but rather they are methods with which we had success. We further compare these results with two univariate methods: a simplification of the previously described MCD by applying it univariately (uMCD) [34], and the commonly used univariate boxplots [22]. Further, both stem from the distance-based outlier methods described for multivariate detection.

Herein, we describe a data quality evaluation process that emphasizes the use of multiple multivariate techniques, communication, and visualization, and assess the sensitivity of standard univariate techniques to detect errors that were identified by multivariate outlier techniques. We describe motivation for, performance and limitations of our process for two exemplar datasets from ONDRI’s vascular cognitive impairment (VCI) cohort, i.e., the neuropsychology and gait platform data at baseline, and provide a roadmap for large or complex datasets, and especially other large-scale projects.

## Methods

### Data

ONDRI consists of 520 participants from multiple sites across Ontario, Canada who have been diagnosed with one of five neurodegenerative diseases: Alzheimer’s disease or amnestic mild cognitive impairment, amyotrophic lateral sclerosis, frontotemporal lobar degeneration, Parkinson’s disease, or vascular cognitive impairment (VCI). Each participant completes baseline assessments at the time of enrolment, and is subsequently followed for up to 3 years to monitor disease progression. Disease characteristics are measured with multiple assessment platforms including neuropsychology, gait, genomics, eye tracking, retinal imaging, and multiple neuroimaging assessments. Participants are required to complete each assessment platform, so individuals with significant cognitive (e.g., Montreal Cognitive Assessment score below 18) or physical (e.g., unable to ambulate independently) impairment are not included. All ONDRI participants provide written and informed consent. Details on the ONDRI study design have been provided previously [13].

In this paper, we consider a core subset of the neuropsychology and gait measurements of the baseline data for the VCI cohort for illustrative purposes. The VCI cohort includes 161 participants between 55 and 85 years of age who experienced an ischemic stroke, as documented by magnetic resonance imaging or computed tomography, at least 3 months prior to enrolment.

The core neuropsychology dataset has 53 variables from 13 cognitive assessments and two questionnaires. Approximately half of the variables within this dataset are raw observed scores, while the other half are the same raw scores transformed based on published education- and/or age-adjusted normative data [35]. As a result, there is strong correlation between the raw and corresponding transformed scores.

The core gait dataset has 29 variables that describe walking performance over multiple experimental conditions (i.e., preferred walking, three types of dual task walking (walking while counting backwards from 100; naming animals; and subtracting multiples of seven from 100), and fast walking) by measuring the number of steps and time required for a 6 metre walk [36]. Depending on the site, the primary source of data collection was through tri-axial accelerometers worn on both ankles and the right hip or an electronic GAITRite walkway, set up across the center portion of the walk [37]. Regardless of the data collection modality, administrators used tablets for tracking the walking condition and recording the time to walk completion, for data verification and in case of equipment malfunction. Unfortunately, the gait data for 13 participants (8.1%) were not properly recorded and these participants are excluded from the analysis as a result, reducing the sample for the gait platform to 148 participants.

Both data platforms performed rigorous QA and QC procedures to maintain accuracy and consistency across multiple testing locations [36]. Extensive training was provided to all study coordinators on all facets of the data collection, including administering, scoring, and entering data into the database.

### Data preparation

We conducted outlier detection on each of the aforementioned datasets with and without adjusting for covariates. From a quality evaluation perspective, adjusting for covariates could illuminate or obscure errors by moving an error away from or towards the observed distribution. Therefore, we investigated whether covariate adjustment altered the outlier and subsequent error results, in comparison to unadjusted versions.

The covariate adjusted dataset was composed of the residuals from a linear regression of each variable with age, sex, years of education, and all interactions. These demographic variables are common adjustment factors for studies of aging or neurodegenerative diseases [38], and further are used in the analysis of many neuropsychology and gait measurements [35, 39]. Summary demographics for the preliminary data for the VCI participants in ONDRI are in Table 1.

**Table 1:** Preliminary summary demographics for the ONDRI VCI cohort

	Neuropsychology	Gait
Sample size	161	148
Age in years, mean (sd)	68.72 (7.42)	68.61 (7.39)
Education in years, mean (sd)	14.61 (2.92)	14.65 (2.98)
Number of Males/Females	110 / 51	104 / 44

For both the adjusted and unadjusted datasets, each measure was scaled to have zero mean and unit standard deviation. This ensures that variables are comparable to one another by preventing scale differences from biasing the results. For multivariate approaches, missing values on any variable would exclude the participant so any missing values were imputed using the univariate mean.

### Notation

Here we define a notation set for use throughout the paper. Upper case bold letters (e.g., **X)** denote matrices and lower case bold letters (e.g., **x)** denote vectors. Two matrices that appear side-by-side (e.g., **XY**) denote standard matrix multiplication. *diag*(**X**) denotes the vector of diagonal elements of matrix **X**, ‖**X**‖_∗_ denotes the nuclear norm (sum of the singular values) of **X**, and ‖**X**‖_1_ is the l_1_-norm of a vectorized **X**. **I** is the identity matrix. The superscript ^−1^ denotes matrix inversion and the superscript ^T^ denotes matrix transpose. Subscripts denote different versions of a matrix or vector (e.g., **x**_**j**_ denotes the *j*^*th*^ column vector of matrix **X**, for *j* = 1, 2, 3, …). Lower case italic letters (e.g., *x*) denote scalar values. ⌊*x*⌋ denotes the floor function. *n* denotes the number of observations, *p* denotes the number of variables, and raw data matrices are assumed to contain *n* × *p* elements.

### Outlier detection methods

We used two multivariate methods to obtain robust estimates and identify multivariate outliers within each dataset: the Minimum Covariance Determinant (MCD) and Candès’ Robust Principal Component Analysis (RPCA). We assessed the sensitivity of the univariate MCD (uMCD) [34] and traditional boxplots [22] to identifying observations originally identified by the multivariate techniques that were found to have errors.

#### Minimum covariance determinant (MCD)

The minimum covariance determinant (MCD) algorithm provides a robust estimate of the multivariate mean $\left(\widehat{\boldsymbol{\upmu}}\right)$ and covariance ($\widehat{\boldsymbol{\Sigma}}\Big)$ by searching for the subset of *h* data points with a minimum determinant of the covariance matrix [34], where $\frac{n+p+1}{2}\le h\le n$. The MCD relies on the Mahalanobis Distance (MD):

$$ \mathbf{m}=\sqrt{{\left(\mathbf{X}-\widehat{\boldsymbol{\upmu}}\right)}^{\mathrm{T}}{\widehat{\boldsymbol{\Sigma}}}^{-\mathbf{1}}\left(\mathbf{X}-\widehat{\boldsymbol{\upmu}}\right)}, $$

Since the covariance cannot be inverted when $\widehat{\boldsymbol{\Sigma}}$ is singular, **m** is undefined for *h* < *p* and thus can be calculated only for datasets with more observations than variables (n > p). To obtain the exact MCD is computationally expensive [34], and so the fast-MCD [40] is used in practice [34].

The fast-MCD begins by randomly selecting a subset of *p* + 1 observations from dataset **X**, producing ${\mathbf{X}}_{\mathbf{0}}^{\ast}$ and subsequently computing **m**_**0**_ for all *n* observations, with mean, ${\widehat{\boldsymbol{\upmu}}}_{\mathbf{0}}$**,** and covariance, ${\widehat{\boldsymbol{\Sigma}}}_{\mathbf{0}}$. The *h* observations for which **m**_**0**_ are smallest are then extracted to form a new subset, ${\mathbf{X}}_{\mathbf{1}}^{\ast}$. *h* is determined by:

$$ h=\left\lfloor 2\left\lfloor \frac{n+p+1}{2}\right\rfloor -n+2\upalpha \left(n-\left\lfloor \frac{n+p+1}{2}\right\rfloor \right)\right\rfloor, $$

where *α* is a user-defined parameter between 0.5 and 1 that specifies the desired robustness, with smaller values equating to increased robustness but at the cost of lower efficiency and a potentially larger set of outliers. From ${\mathbf{X}}_{\mathbf{1}}^{\ast}$, **m**_**1**_ is computed for all *n* observations and a new subset of *h* observations is selected, decreasing the covariance determinant with each new subset. This process repeats until the subset of *h* observations at a given iteration is the same subset as the previous iteration. At this point, the determinant of the covariance is a localized minimum. The algorithm is repeated for every *p + 1* subset, or for some maximum number of random subsets (e.g., 100,000), and the subset of *h* observations with the (global) minimum covariance determinant is defined as the most concentrated subset, hereafter called ${\mathbf{X}}_{\mathbf{MCD}}^{\ast}$. The MCD-robust parameter estimates are subsequently calculated:

$$ {\widehat{\boldsymbol{\upmu}}}_{\mathbf{MCD}}=\frac{1}{h}\sum \limits_{\mathrm{i}=1}^h{{\mathbf{x}}_{\mathbf{MCD}}}_{\mathbf{i}}{\widehat{\boldsymbol{\Sigma}}}_{\mathbf{MCD}}={c}_0\frac{1}{h}\sum \limits_{\mathrm{i}=1}^h\left({{\mathbf{x}}_{\mathbf{MCD}}}_{\mathbf{i}}-{\widehat{\boldsymbol{\upmu}}}_{\mathbf{MCD}}\right){\left({{\mathbf{x}}_{\mathbf{MCD}}}_{\mathbf{i}}-{\widehat{\boldsymbol{\upmu}}}_{\mathbf{MCD}}\right)}^{\mathrm{T}} $$

where *c*_0_ is a scalar consistency factor to correct for the smaller sample [41, 42], followed by the corresponding **m**_**MCD**_ estimates.

To determine the threshold of **m**_**MCD**_ beyond which an observation is considered an outlier, we follow an approach similar to Dovoedo & Chakraborti [43]. First, we transform each **m**_**MCD**_ to the Robust Mahalanobis Distance Outlyingness [43] statistic to constrain the distribution of distances to between zero and one:

$$ {\mathbf{r}}_{\mathbf{MCD}}=1-\frac{1}{1+{\mathbf{m}}_{\mathbf{MCD}}} $$

Then, we simulate 100 multivariate normal samples of size *n* with ${\widehat{\boldsymbol{\upmu}}}_{\mathbf{MCD}}$ and ${\widehat{\boldsymbol{\Sigma}}}_{\mathbf{MCD}}$, calculate the outlyingness of the simulated observations, and use the *ϵ*_*MCD*_ percentile from these simulations as the threshold for outliers, where *ϵ*_*MCD*_ is user-defined and between 0 and 1.

Like most multivariate outlier techniques, there is no direct information about the source of outlyingness for each observation. Therefore, we supplement the MCD with the corr-max transformation [33]. The corr-max transformation identifies a partition, **W**, that breaks the **m**_**MCD**_ into a sum of *p* terms, where the combination of variables that contribute most to the sum are those that most influence the value of **m**_**MCD**_**,** i.e., ${\mathbf{m}}_{\mathbf{MCD}}^{\mathbf{2}}=\sum \limits_{\mathrm{j}=1}^{\mathrm{p}}{{\widehat{\mathbf{w}}}_{\mathbf{j}}}^{\mathbf{2}}$. To identify this partition, we obtain the transformation matrix:

$$ \widehat{\mathbf{C}}={\left({\widehat{\mathbf{D}}}_{\mathbf{MCD}}{\widehat{\boldsymbol{\Sigma}}}_{\mathbf{MCD}}{\widehat{\mathbf{D}}}_{\mathbf{MCD}}\right)}^{-\frac{\mathbf{1}}{\mathbf{2}}}{\widehat{\mathbf{D}}}_{\mathbf{MCD}}, $$

where ${\widehat{\mathbf{D}}}_{\mathbf{MCD}}$ is a diagonal matrix with entries $1/\sqrt{\mathit{\operatorname{diag}}\left({\widehat{\boldsymbol{\Sigma}}}_{\mathbf{MCD}}\right)}$. Finally, the contribution matrix is derived by multiplying $\widehat{\mathbf{C}}$ with the vector of deviations from the mean, ${\mathbf{x}}_{\mathbf{i}}-{\widehat{\boldsymbol{\upmu}}}_{\mathbf{MCD}}$. The contribution matrix is proportional to the identity matrix, and the maximum of the sum of correlations between each variable’s observed values and corresponding vector of contributions is obtained. As a result, a deviation of a given magnitude will have a larger measured contribution on variables with less noise, versus a smaller contribution on variables with more noise. The resulting contribution values highlight variables that contribute the most variance to each robust MD, and can be used as a guide for determining the reason each outlier is considered atypical.

#### Robust principal component analysis (RPCA)

Candès’ Robust Principal Component Analysis (RPCA) [32] aims to deconstruct a dataset **X** into two separate datasets, **L** and **S**, where **X = L** + **S**. Here **L** is a low-rank and robust approximation of **X**, and **S** is a generally sparse matrix with non-zero values representing deviations from the robust structure. **L** and **S** are determined by optimizing:

$$ \min \left\{{\left\Vert \mathbf{L}\right\Vert}_{\ast }+\lambda {\left\Vert \mathbf{S}\right\Vert}_1\right\}\kern0.5em \mathrm{s}.\mathrm{t}.\mathbf{X}=\mathbf{L}+\mathbf{S}, $$

where *λ* is a user-defined regularization parameter that controls the level of sparsity in **S,** commonly defined as $\lambda =\frac{1}{\sqrt{\max \left(n,p\right)}}$. However, **S** often contains many non-zero values, and so we have introduced a user-defined hyperparameter, *τ*, to allow the user to specify more directly the proportion of non-zero elements desired (e.g., *τ* = 5%). In practice, RPCA can be performed in the following five steps:

1. Set ${\mathbf{X}}_{\mathbf{0}}^{\prime }=\mathbf{X}-{\mathbf{S}}_{\mathbf{0}}^{\prime }$, where ${\mathbf{S}}_{\mathbf{0}}^{\prime }$ is a *n* × *p* matrix of zeros.
2. Apply the Singular Value Decomposition [29] such that ${\mathbf{X}}_{\mathbf{0}}^{\prime }={\mathbf{U}}_{\mathbf{0}}{\boldsymbol{\Delta}}_{\mathbf{0}}{\mathbf{V}}_{\mathbf{0}}^{\mathbf{T}}$, and threshold **Δ**_**0**_ to ${\boldsymbol{\Delta}}_{\mathbf{0}}^{\prime }$ through a regularization procedure such as [44].
3. Construct $$ {\mathbf{L}}_{\mathbf{0}}={\mathbf{U}}_{\mathbf{0}}{\boldsymbol{\Delta}}_{\mathbf{0}}^{\prime }{\mathbf{V}}_{\mathbf{0}}^{\mathbf{T}} $$.
4. Compute $$ {\mathbf{S}}_{\mathbf{0}}={\left\Vert {\mathbf{X}}_{\mathbf{0}}^{\prime }-{\mathbf{L}}_{\mathbf{0}}\right\Vert}_1 $$.
5. If ${\mathbf{X}}_{\mathbf{0}}^{\prime }-{\mathbf{L}}_{\mathbf{0}}-{\mathbf{S}}_{\mathbf{0}}\approx 0$, stop. Else return to step 2 with ${\mathbf{X}}_{\mathbf{1}}^{\prime }={\mathbf{L}}_{\mathbf{0}}+{\mathbf{S}}_{\mathbf{0}}$ .

For our work, the five steps above are repeated, decreasing *λ* each time until the proportion of non-zero elements are <*τ*.

To assess the deviation of participants and determine which are outlying, we supplement the RPCA by measuring the Orthogonal Distance (OD), that is, the sum of squared distance between two datasets. This was motivated by Hubert, Rousseeuw, & Vanden Branden’s use of the OD [45] to identify observations that change substantially between two versions of the same data. We calculate the OD between **X** and **S**:

$$ {\mathbf{o}}_{\mathbf{XS}}=\sum \limits_{\mathrm{j}=1}^p{\left({\mathbf{x}}_{\mathbf{j}}-{\mathbf{s}}_{\mathbf{j}}\right)}^2 $$

and similarly for **L** and **S**, **o**_**LS**_. For each participant, the proportion between **o**_**XS**_ and **o**_**LS**_ represents the deviation between the robust estimates and observed values, given the sparsity in **S**. To determine which participants are outliers, the proportional OD estimates are ordered and the *ϵ*_*RPCA*_ percentile is used as a threshold. The non-zero values in **S** for each outlier are considered the contributing variables for the RPCA approach, and the proportion dictates the order of importance.

#### Univariate MCD

The univariate MCD (uMCD) generally follows the same process as the previously described MCD, but with some simplifications. Since the approach is applied to each variable independently, the subset of *h* concentrated data points is selected based on the variance, and the robust estimates are the mean and variance [34]. The Monte Carlo simulations of a multivariate normal distribution were replaced with simulations of a normal distribution.

#### Boxplots

Boxplots use the median and the two other quartiles as measures of location and spread, as opposed to the mean and standard deviation, and are consequently more robust by design. To identify outliers, the interquartile range is calculated as the difference between the first and third quartiles, and any values greater than 1.5 times the interquartile range from the third quartile, or more than 1.5 times the interquartile range less than the first quartile, are considered outlying. Visually, these are represented as any values beyond the whiskers. Although the boxplot is often regarded as an informal or exploratory approach to outlier detection [28, 47], is it one of the simplest methods to identify atypical observations on the univariate scale, is not affected by the magnitude of outliers, and offers clear visualization.

### List of outliers

It is expected that each outlier detection method will produce a different subset of outliers due to the differences in technique. Further, while the multivariate methods we used consider patterns between variables when identifying outliers, they also note participants that are extreme on a single variable (i.e., univariate outliers) and therefore remain valuable when variables are fully uncorrelated. As a result, we did not include univariate techniques in our initial assessment of outliers and therefore only considered the results of the multivariate approaches when selecting participants to review for accuracy.

Ultimately, participants identified as outlying with two or more of the four multivariate approaches (i.e., MCD or RPCA; adjusted or unadjusted) were compiled into a single list, based on the assumption that outliers detected at higher frequencies are more generally deviant. The contributing variables were also included to guide the platforms in determining the location of potentially erroneous recorded observations. This list is forwarded to the corresponding platform team, where the recorded data for each outlying observation are verified and subsequently classified as erroneous or atypical but correct. Values with evidence that an error occurred are corrected, while truly outlying values are left unchanged and accommodated during the analysis phase as indicated by the specific goals of the researcher. Note that the selected outliers and proposed reasons for detection are based on thresholds jointly chosen by the biostatistics and assessment platform teams. Therefore, data highlighted in the overall list should be approached with curiosity, as they can change with additional iterations of outlier detection.

### Multivariate visualization

With univariate methods, understanding why a participant is flagged is straightforward as the variable on which the participant is deviating is clear, and many common visualization techniques, such as boxplots and histograms, can be used. While identifying contributing variables for each outlying participant allows us to better pinpoint why they were flagged, these lists can be difficult to appreciate without also understanding the observation’s relation to the rest of the data structure. Therefore, multivariate visualization was used to gain some understanding of how each outlying participant deviates. Specifically, the observed values of all participants were mapped on a scatterplot for the two primary contributing variables of a given outlying participant. Where the reason for outlying did not become obvious, a colour or size gradient was used to depict a third variable.

### The data quality evaluation process

**Fig. 1:**
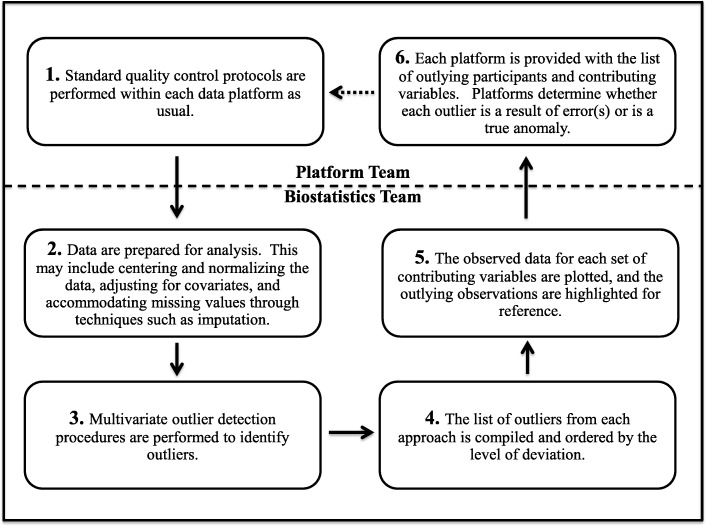
The data quality evaluation process steps, represented as a process that may loop. The dashed line separates steps that are performed by the platform team from the biostatistics team, while the dashed arrow indicates that the process will not always return to Step 1

We have described the pieces of our proposed data quality evaluation process. Figure 1 summarizes the steps for an iterative process that other large-scale projects can employ as part of their own data quality evaluation to identify outliers and potential errors.

A new iteration of this process should occur each time an error is identified, prompting corrections and generation of a new dataset, as corrected data may alter the set of outlying observations. When none of the outlying participants are determined to be an error and the dataset remains static, the data quality evaluation process is considered complete.

In order to perform these multivariate outlier detection procedures efficiently, an R package *outlieRs* [48] was developed. This package is available online for use by other large-scale projects.

## Results

Our proposed data quality evaluation process was applied to the neuropsychology and gait core datasets. In this section, we provide results for each of the datasets from four multivariate approaches – MCD or RPCA, with or without covariate adjustment – and assess the sensitivity of four univariate approaches – univariate MCD or boxplots, with or without covariate adjustment – to the errors identified.

### Outliers and errors

Parameters were selected for each of the multivariate outlier detection approaches based on the number of observations and variables in the dataset, the data distribution, the resources that would be required to verify the resulting outlier list within platforms, and the unknown relative performance characteristics of the MCD and the RPCA. The same parameters for both the neuropsychology and gait datasets were used in order to compare results between different datasets: *α* = 0.8 and *ϵ*_*MCD*_ = 0.99 were used for the MCD, and *λ* = $\frac{1}{\sqrt{\max \left(n,p\right)}}$, *ϵ*_*RPCA*_ = 0.90, and *τ* = 0.05 were used for the RPCA. The impact of varying *α* and *ϵ*_*RPCA*_ on the number of outliers for each approach, and how a change would affect the detection of errors, is explored in Additional file 1. For the univariate approaches, the parameters for the uMCD were set equal to those selected for the MCD (*α* = 0.8 and *ϵ*_*MCD*_ = 0.99) for a direct comparison.

Table 2 shows the number of participants identified as outliers by multivariate methods. The summary compares the number of outlying participants identified with the two methods, regardless of whether covariates were adjusted, while the individual results compare adjusted and unadjusted within each method. The numbers of errors that were identified after verifying the values of participants identified by two or more approaches are also compared.

**Table 2:** Comparison of outlying participants and errors identified by the multivariate outlier detection approaches during the first iteration of the data evaluation process: first, between MCD and RPCA directly, combining results with and without adjustment; then, between the adjusted and unadjusted results within each multivariate method. For each set of results, the total number of outliers/errors by each approach is reported (MCD vs. RPCA; adjusted vs. unadjusted), as well as the number that overlapped between the two approaches

	Neuropsychology *n* = 161; *p* = 53			Gait *n* = 148; *p* = 29		
Summary	MCD & RPCA	MCD	RPCA	MCD & RPCA	MCD	RPCA
(Adj. & Unadj. are combined)						
Outlying Participants	11	26	29	19	29	33
Number of Errors	6	8	6	3	5	3
Individual Results	Adj. & Unadj.	Adj.	Unadj.	Adj. & Unadj.	Adj.	Unadj.
MCD						
Outlying Participants	18	22	22	24	25	28
Number of Errors	8	8	8	5	5	5
RPCA						
Outlying Participants	16	22	23	19	26	26
Number of Errors	4	4	6	3	3	3

Univariate outlier detection methods were performed to assess whether the errors identified with multivariate methods could have been identified with simpler methods. The number of outlying participants and errors identified by univariate methods are in Additional file 2. Table 3 compares results between multivariate and univariate methods, regardless of adjustment. Note that while participants with an error may have been identified with a univariate approach, we only included them as errors if the corresponding variable was flagged. Further, only participants flagged with two or more multivariate methods were verified, so we are not able to report errors identified by only univariate approaches.

**Table 3:** Comparison of outlying participants and errors identified by the multivariate and univariate outlier detection approaches in the first iteration of the data evaluation process, regardless of specific method and whether covariate adjustment was applied

	Neuropsychology *n* = 161; *p* = 53			Gait *n* = 148; *p* = 29		
	Multi. & Uni.	Multi.	Uni.	Multi. & Uni.	Multi.	Uni.
Outlying Participants	44	44	133	25	43	42
Number of Errors	3^a^	8	3^b^	3	5	3^b^

^a^All outlying participants identified by multivariate methods were also identified by univariate methods. However, not all univariate methods identified the participant as an outlier on the variable with the error

^b^Outlying participants identified by univariate methods only were not verified.

#### Neuropsychology

On the first iteration of the quality evaluation process, 44 participants (27.3%) were identified by one or more of the four multivariate outlier approaches across 53 neuropsychology variables. The MCD identified 22 participants (13.7%) with each of the adjusted and unadjusted approaches, and the RPCA identified 22 participants (13.7%) with the adjusted and 23 participants (14.3%) with the unadjusted approach; 11 participants (6.8%) were identified with both the MCD and the RPCA, regardless of adjustment.

The list provided to the neuropsychology team for verification comprised the 29 participants (18.0% of all 161 participants) that were flagged by two or more multivariate approaches. For each of the listed participants, the neuropsychology team compared the values stored in the database with those recorded on the paper form (how the data were originally collected) and, where possible, with the audio recordings of the participant’s responses. Values differed between the recorded and source data for eight of the 29 participants (27.6%) on the list, 5.0% of all 161 participants. The MCD was successful in identifying all eight of these participants, while the RPCA was successful in identifying six (75.0%).

Using the contributing variables identified by each of the approaches proved valuable in highlighting the variables upon which to focus. With the MCD, the erroneous variable was among the top three contributing variables for seven of the eight participants with errors, regardless of adjustment, while the erroneous variables were included among the set of contributing variables for four of the six participants with errors that the RPCA identified. The error identified despite the variable not having been a primary contributor highlights the idea that multivariate outliers are the result of relationships among all the variables, not only the first few contributors, and atypical relationships may be hiding deeper.

With univariate outlier detection, all values within the subset identified by the uMCD were equal for one variable when covariate adjustment was not applied, resulting in a robust variance of 0 and making it impossible to calculate the Mahalanobis distance. As a result, this variable was excluded from the unadjusted approach. Nevertheless, 133 of the 161 participants (82.6%) were identified by at least one approach on at least one variable. One hundred twenty participants (74.5%) were flagged by the uMCD with covariate adjustment, 126 participants (78.3%) were identified by the uMCD unadjusted approach, 80 participants (49.7%) were flagged by boxplots with adjustment, and 78 participants (48.4%) were flagged by boxplots without adjustment. We note that all 44 participants identified by the multivariate approaches were also identified by the univariate approaches on at least one variable.

**Fig. 2:**
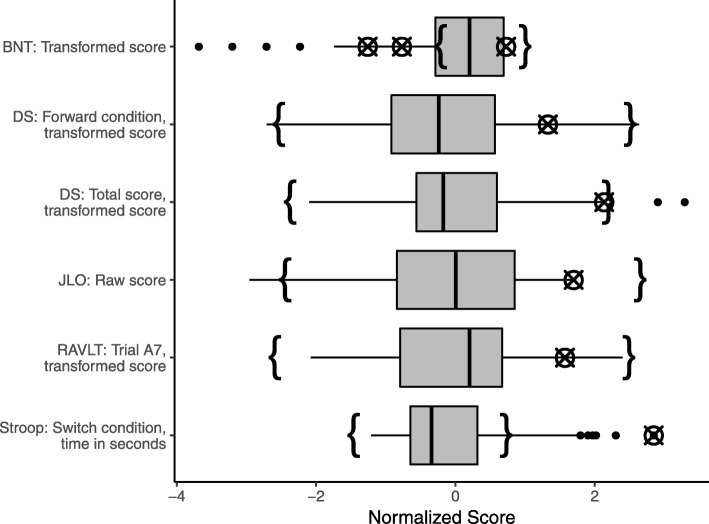
Boxplots for neuropsychology variables on which an error was identified with the multivariate data quality evaluation process. All data were adjusted for age, sex, and years of education, and normalized to have zero mean and unit standard deviation. The range of typical values identified by the univariate MCD is represented by curly brackets. Values at which an error was identified with the data quality evaluation process are represented by crossed circles. BNT = Boston Naming Test. DS = Digit Span assessment. JLO = Judgement of Line Orientation. RAVLT = Rey Auditory Verbal Learning Test. Stroop = Colour-Word Interference

While the univariate approaches were not considered when building the list, we confirmed post hoc that using only the uMCD would have yielded only two or three of the errors identified with multivariate methods, depending on adjustment, while the boxplots would have yielded only one. Figure 2 shows boxplots for the variables on which errors were identified with the multivariate approaches, not adjusted for covariates. The crossed circles on each boxplot represent the erroneous values within the univariate distribution, and the curly brackets represent the non-outlying range identified by the uMCD, showing a similar span to the whiskers on the boxplots, though occasionally narrower or shifted, causing an increase in values identified as outliers. Most errors that were not identified using either univariate method were errors from manually calculating transformed scores from raw scores, and so were only evident when both types of scores were considered together.

After correcting the eight identified errors, the entire data evaluation process was performed again. This subsequent iteration of the process identified four additional errors in the data that had not been identified during the first pass. Most intriguingly, one of the participants with an error identified in the initial iteration was identified again in the second iteration – while the initial error for this participant had been corrected, the second iteration enabled a different error to become apparent. The newly identified error had been masked during the first iteration by the original error, thus validating the use of an iterative process until errors are no longer identified and the list of outliers remains static.

#### Gait

On the first iteration of the quality evaluation process, 43 participants (29.1%) were identified by at least one of the multivariate outlier approaches. The MCD identified 25 participants (16.9%) with the adjusted and 28 participants (18.9%) with the unadjusted approach, and the RPCA identified 26 participants (17.6%) with each of the adjusted and unadjusted approaches; 19 participants (12.8%) were identified by both the MCD and RPCA, regardless of adjustment.

The list provided to the gait team for verification comprised the 29 participants (19.6% of all 148 participants) that were identified by two or more multivariate approaches. For each of the listed participants, the gait team reprocessed the data using participant-specific thresholds for step detection and manually inspected each footstep. Through the manual inspection, they discovered erroneous step counts were calculated by the software for five of the 29 participants (17.2%) on the list, or 3.4% of all 148 participants. Again, the MCD was successful in identifying all five of these participants, and the RPCA identified three (60.0%).

The contributing variables were again effective in pointing towards the source of error, but unlike with the neuropsychology data, they did not indicate all variables affected by an error. The errors identified in the gait dataset were as a result of the pre-processing pipeline miscounting the number of steps taken by the participant during the walk, and as a result affected all values for that walk. Therefore, while many of the contributing variables possessed an error, they were only those that appeared most atypical as a result of the error and were not the only values affected. In order to pinpoint these errors, the gait expert was required to examine data beyond the top contributing variables.

With univariate outlier detection, 42 of the 148 participants (28.4%) were identified by at least one approach on at least one variable. Thirty-eight participants (25.7%) were flagged by the uMCD with covariate adjustment, 35 participants (23.6%) were flagged by the uMCD without adjustment, 29 participants (19.6%) were identified by boxplots with adjustment, and 26 participants (17.6%) were flagged by boxplots with the unadjusted approach. In total, 25 participants (16.9%) identified by at least one multivariate approach were also flagged by a univariate approach on any variable.

**Fig. 3:**
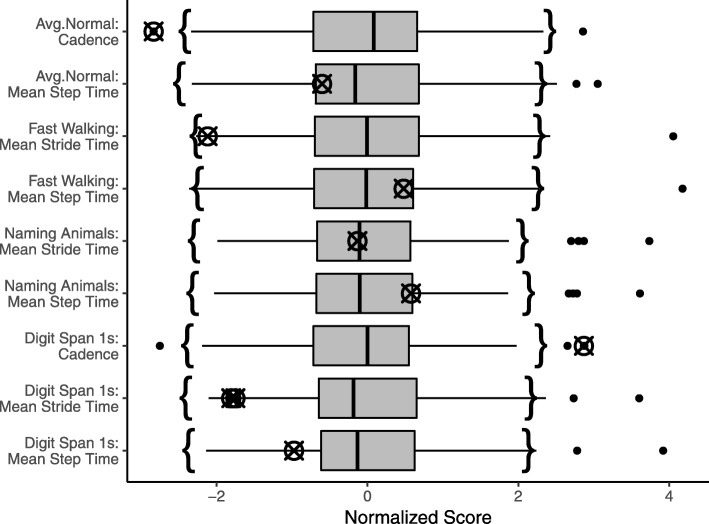
Boxplots for gait variables identified as primary contributing variables and on which an error was identified with the multivariate data quality evaluation process. All data were adjusted for age, sex, and years of education, and normalized to have zero mean and unit standard deviation. The range of typical values identified by the univariate MCD is represented by curly brackets. Values at which an error was identified with the data quality evaluation process are represented by crossed circles. As previously noted, errors identified in the gait dataset affected multiple variables, so two variables are included per error

Again, while the univariate approaches were not considered when building the list, it was confirmed post hoc that using only a univariate method would have missed some of the errors the multivariate approaches were able to find. In particular, the univariate approaches identified only two or three of the errors, depending on adjustment. Figure 3 shows boxplots for the contributing variables identified by the multivariate outlier detection approaches, with data not adjusted for covariates. Again, the crossed circles represent the erroneous values within each univariate distribution, and the curly brackets represent the non-outlying range identified by the uMCD. In this case, the uMCD range was almost identical to the span of the whiskers, suggesting that it would not be possible for any univariate outlier detection method to identify some of the errors the multivariate approaches could.

Subsequent iterations of the data quality evaluation process did not reveal any additional errors in the gait dataset.

### Multivariate visualization

**Fig. 4:**
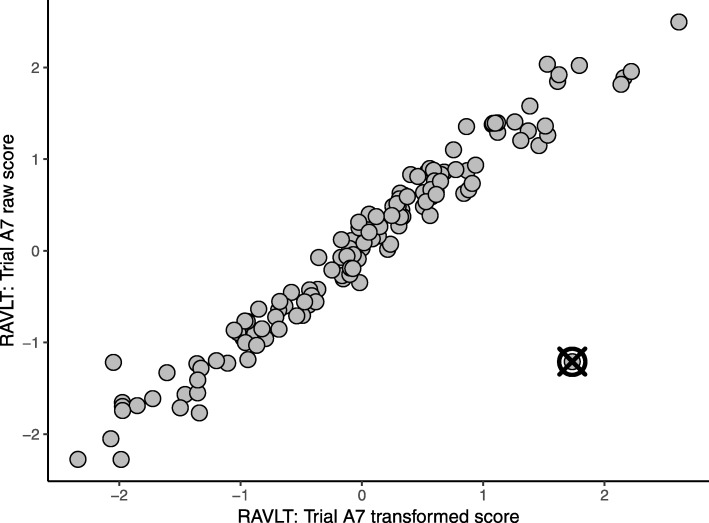
Observed data for two measures of the Rey Auditory Verbal Learning Test (RAVLT). All data were adjusted for age, sex, and years of education, and normalized to have zero mean and unit standard deviation. The outlier is represented by a crossed circle

Visualization proved to be an effective diagnostic and communication tool as it allows viewing how each outlying participant deviated from the others. Figure 4 exemplifies a scatterplot of the top two contributing variables for an outlying participant. This representation clearly conveys that there is a correlation between the raw and transformed scores for the A7 trial of the Rey Auditory Verbal Learning Test [35], with the exception of a single participant whose transformed score was considerably higher than that of other participants with a similar raw score. This participant appears typical on each variable individually (as exhibited by the boxplot in Fig. 2) and consequently would not be easily identified without the benefit of the scatterplot which illustrated the bivariate relationship for these variables. As a result, the plots assisted the biostatistics team in motivating the utility of multivariate outlier detection to platforms.

**Fig. 5:**
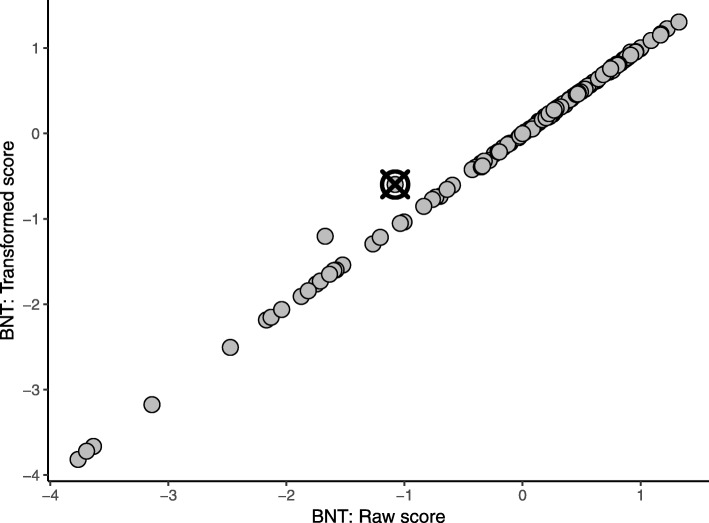
Observed data for two measures of the Boston Naming Test (BNT). All data were adjusted for age, sex, and years of education and normalized to have zero mean and unit standard deviation. The outlier is represented by a crossed circle

**Fig. 6:**
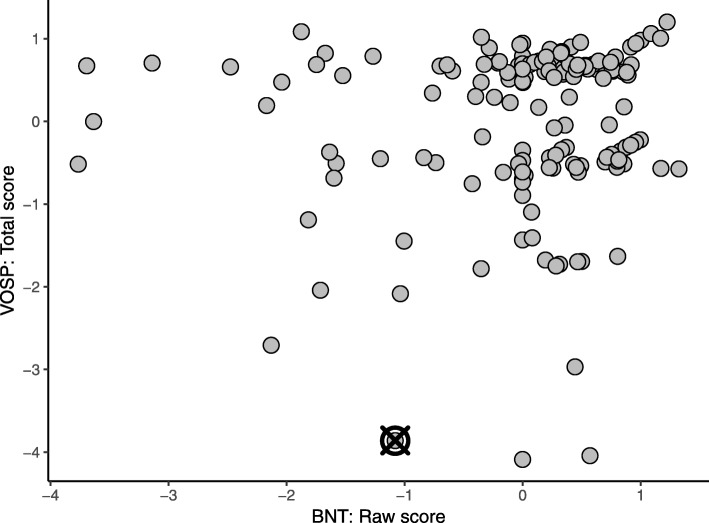
Observed data for a measure from each of the Boston Naming Test (BNT) and the Visual Object and Space Perception battery (VOSP). All data were adjusted for age, sex, and years of education, and normalized to have zero mean and unit standard deviation. The outlier is represented by a crossed circle

Visualization also highlighted the differences between the MCD and RPCA. Data for two primary contributing variables selected by each method are plotted in Figs. 5 and 6. Observe that the data for contributing variables identified with the MCD, plotted in Fig. 5, exhibit a 1:1 relationship, with the exception of two observations with slightly higher transformed BNT scores given their raw BNT scores. Both of these participants were flagged as outliers and identified as erroneous on the transformed BNT score, however only the participant marked by the crossed circle was also identified by the RPCA. Further, the contributing variables selected by the RPCA did not include the transformed BNT score but a Digit Span variable instead. With this difference, the patterns exhibited by the contributing variables identified with the RPCA do not convey a strong correlation like the MCD, as shown in Fig. 6, and the outlying participant is merely away from the cluster formed by the bulk of the observations. While this is also a valid outlier, the erroneous value for the transformed BNT score was not flagged.

## Discussion

The data quality evaluation process proved effective in identifying errors in each of the neuropsychology and gait datasets. The identification of thirteen errors across both datasets on the first iteration of the process shows that typical manual curation is insufficient, and both teams revisited their processes after these results. Further, multivariate approaches are necessary as seven of the thirteen errors (53.8%) were not flagged as outliers by univariate methods, and were apparent only when multiple variables were considered together.

Identifying extreme values on each variable independently is important, and it can be argued that univariate outlier detection is an important step to perform in its own right. However, we assumed that since multivariate methods identify points located away from the cluster center, not merely those with atypical relationships, that performing univariate detection separately would be redundant. Further, verification of participants and values identified using univariate approaches as well would have increased the outlier lists. Particularly, for the neuropsychology dataset, the list would have increased from 29 to at least 113 of 161 participants, given the overlap and large number of outliers in adjusted and unadjusted uMCD, even if the rule of verifying participants identified with two or more approaches remained. Given the time and effort involved in the iterative error detection process, we considered testing up to 70% of participants for errors to be impractical and beyond our resources. Further, while the univariate thresholds were quite liberal and could have been made more stringent, many errors identified with the multivariate approaches were missed with the liberal thresholds nonetheless, so reducing the number of outliers detected would seem less optimal.

Based on communication between the biostatistics and data platform teams, it was deduced that sources of error included both human and technological mistakes. Specifically, errors in the neuropsychology dataset occurred as a result of incorrectly recording values in the database or incorrectly computing transformed scores manually, while errors in the gait dataset occurred as a result of the processing script misidentifying the number of steps made by the participant. Further, because many of the calculated variables for a walking task are dependent on the total number of steps recorded, an error in identifying the number of steps subsequently made the other measures for that task erroneous, and multiple values had to be recalculated for each of the affected participants.

Each of the four multivariate outlier detection approaches identified slightly different subsets of outliers, and the overlap and differences between them allowed us to better understand aspects of these methods. Consider the overlap between the MCD and the RPCA, regardless of adjustment: 11 of the 44 participants (25.0%) flagged in the neuropsychology dataset were identified with both methods, while 19 of the 43 participants (44.2%) flagged with the gait dataset were identified with both methods. While we could assume that these overlaps highlight participants that are more outlying, it begs to be asked why other participants were identified with only one method. One possible explanation is offered by Figs. 5 and 6: that each method is more successful at identifying outliers with a specific type of deviation. Specifically, the contributing variables identified by the MCD suggest that the MCD is more sensitive when strong correlations exist, while the RPCA is less influenced by these correlations.

Manual QA and QC procedures occurred within each data platform, but the characteristics of any observations that were identified as erroneous (and subsequently corrected) at that stage were not recorded. However, the MCD had better success in identifying both errors and the contributing variables for errors than the RPCA in both the neuropsychology and gait datasets. Further, the erroneous observations missed by the RPCA typically possessed characteristics similar to those in Fig. 5, suggesting that errors of this nature are more difficult to identify with typical QC procedures.

Finally, if the MCD and the RPCA are effectively focusing on different types of outliers, it may not be appropriate to compile an outlier review list based on the frequency of identification, but to give more weight to visualization. This is especially true because two errors in each of the neuropsychology and gait datasets were not identified with the RPCA, regardless of adjustment, and therefore would not have been verified had the overall list been limited to participants identified by three or more approaches, as opposed to two. Nevertheless, we do not propose that the MCD and RPCA are the most appropriate multivariate outlier detection techniques, and encourage the reader to explore some of the many other approaches available (see [18, 46, 49, 50] for reviews).

While there is often a desire for an objective decision making process in statistics [51], knowledge about the subject field and data generation processes are also critical in order to make appropriate decisions. In fact, it has been argued that outliers identified by automated algorithms should be thought of as *potential* outliers and that field experts should decide which nominations require accommodation [23].

### Limitations

These methods are data dependent and identify observations that deviate from patterns established by other observations. Therefore, while there was success in identifying previously unknown errors, erroneous observations that did not deviate from the pattern were not identified through this process. Further, systematic errors (i.e., those that occur as a result of an incorrect operation that invalidates all or a subset of the observed values, such as a protocol deviation) are also unlikely to be identified if a sufficient number of observations with the systematic error exist. Therefore, traditional quality assurance and quality control procedures should continue to be applied a priori, as errors will distort the true pattern and may dominate the distribution.

The MCD and the RPCA are limited to datasets with exclusively continuous data. As a result, some categorical variables were excluded from the data quality evaluation process, leading to the possibility that errors may have been missed. This will be especially limiting in platforms with a high proportion of categorical variables (e.g., genomics). Therefore, extending the MCD and the RPCA for use with categorical and mixed data types is an area in which future research should be focused [52].

Finally, the MCD is undefined for *n* < *p*, as previously mentioned, and so the RPCA must be used with wide datasets. While some amendments have been proposed to address this limitation in the MCD [45, 53], we cannot comment on their effectiveness in relation to the RPCA.

## Conclusions

We have implemented a process that effectively identifies erroneous observations using multivariate outlier detection techniques in two exemplary datasets from different data platforms of ONDRI. This framework has facilitated the improvement of data integrity as errors can be corrected, and a new dataset can be generated prior to analysis. It was imperative that multivariate approaches were used as univariate methods missed errors that exist only when multiple variables were considered together. In particular, the MCD proved to be more effective as the RPCA consistently missed errors that the MCD identified, though cannot be used for datasets with fewer observations than variables.

## Additional files

Additional file 1: Effect of selected parameters. (DOCX 17 kb)

Additional file 2: Comparison of outlying participants and errors identified by the univariate outlier detection approaches during the first iteration of the data evaluation process: first, between uMCD and boxplots directly, combining results with and without adjustment; then, between the adjusted and unadjusted results within each univariate method. For each set of results, the total number of outliers/errors by each approach is reported (uMCD vs. Boxplots; adjusted vs. unadjusted), as well as the number that overlapped between the two approaches. (DOCX 13 kb)

## Abbreviations

ADNI: Alzheimer’s Disease Research Initiative
CLSA: Canadian Longitudinal Study on Aging
MCD: Minimum Covariance Determinant
MD: Mahalanobis Distance
OD: Orthogonal Distance
ONDRI: Ontario Neurodegenerative Disease Research Initiative
PCA: Principal Component Analysis
PPMI: Parkinson’s Progression Markers Initiative
QA: Quality assurance
QC: Quality control
RPCA: Robust Principal Component Analysis
VCI: Vascular cognitive impairment
