# Supplementary material for: The utility of multivariate outlier detection techniques for data quality evaluation in large studies: an application within the ONDRI project
Source: BMC Med Res Methodol. 2019 May 15;19:102. doi: 10.1186/s12874-019-0737-5 (PMC6521365; doi:10.1186/s12874-019-0737-5)
Supplement: Supplementary file 1 — Effect of selected parameters. (DOCX 17 kb) [file 12874_2019_737_MOESM1_ESM.docx]

**Additional file 1: Effect of Selected Parameters**

Selected parameter values for MCD and RPCA were those that proved effective for our data given the number of observations and variables in each dataset, with the objective of producing a manageable outlier review list given the capacity of the data teams verifying the recorded observations. Since each data platform team was given a specific outlier list from which these errors were identified, we are not able to say how many additional errors may have been identified had the list been longer. However, we can adjust parameters *post hoc* to determine how they affect the number of outliers and known errors identified. Therefore, we report on values of 0.75, 0.85, and 0.9 for $\alpha$ and values of 0.85, 0.95, and 0.99 for $\epsilon_{RPCA}$, as these are the parameters that affect the robust structure identified in the MCD and RPCA, respectively. Table S1 shows the number of outliers and known errors that would have been identified with different parameters in comparison to the parameters chosen originally.

**Table S1: Outliers and known errors identified with varying parameters. Bold font indicates results using the originally chosen parameters**

| Dataset | Covariate Adjustment | Outlier Method | Parameter Selection | Number of Outliers | Number of Known Errors |
| --- | --- | --- | --- | --- | --- |
| Neuropsychology | Adjusted | MCD | 0.75 | 27 | 8 |
|  |  |  | **0.8** | **22** | **8** |
|  |  |  | 0.9 | 18 | 8 |
|  |  |  | 0.95 | 21 | 8 |
|  |  | RPCA | 0.85 | 36 | 5 |
|  |  |  | **0.9** | **22** | **4** |
|  |  |  | 0.95 | 11 | 2 |
|  |  |  | 0.99 | 3 | 1 |
|  | Unadjusted | MCD | 0.75 | 27 | 8 |
|  |  |  | **0.8** | **21** | **8** |
|  |  |  | 0.9 | 17 | 8 |
|  |  |  | 0.95 | 23 | 8 |
|  |  | RPCA | 0.85 | 36 | 6 |
|  |  |  | **0.9** | **23** | **6** |
|  |  |  | 0.95 | 13 | 3 |
|  |  |  | 0.99 | 2 | 0 |
| Gait | Adjusted | MCD | 0.75 | 30 | 5 |
|  |  |  | **0.8** | **25** | **5** |
|  |  |  | 0.9 | 26 | 5 |
|  |  |  | 0.95 | 25 | 5 |
|  |  | RPCA | 0.85 | 41 | 3 |
|  |  |  | **0.9** | **26** | **3** |
|  |  |  | 0.95 | 13 | 2 |
|  |  |  | 0.99 | 3 | 0 |
|  | Unadjusted | MCD | 0.75 | 30 | 5 |
|  |  |  | **0.8** | **28** | **5** |
|  |  |  | 0.9 | 27 | 5 |
|  |  |  | 0.95 | 27 | 5 |
|  |  | RPCA | 0.85 | 40 | 4 |
|  |  |  | **0.9** | **26** | **3** |
|  |  |  | 0.95 | 13 | 2 |
|  |  |  | 0.99 | 3 | 0 |

The MCD and the RPCA were affected differently by adjusting these parameters. For all values of $\alpha$, the sets of outliers identified with the MCD were approximately equal in size, varying by only a few outliers in most cases. It should be noted, however, that sets of the same size were not necessarily composed of the same participants in entirety as some participants moved above or below the outlier threshold with changes in $\alpha$ and subsequent changes in the concentrated subset. Nevertheless, the known errors were always identified. On the other hand, the number of outliers identified with the RPCA was affected by the value of $\epsilon_{RPCA}$, identifying fewer outliers with higher values. Further, the detection of known errors was proportional to the number of outliers, so, increasing the parameter value would have reduced the size of the outlier set and excluded some of the known errors from detection. Contrarily, using lower parameter values would have allowed known errors missed by the RPCA at our chosen parameters to be identified, though it would have been at the cost of a larger set of outliers requiring verification.
