# Supplementary material for: The utility of multivariate outlier detection techniques for data quality evaluation in large studies: an application within the ONDRI project
Source: BMC Med Res Methodol. 2019 May 15;19:102. doi: 10.1186/s12874-019-0737-5 (PMC6521365; doi:10.1186/s12874-019-0737-5)
Supplement: Supplementary file 2 — Comparison of outlying participants and errors identified by the univariate outlier detection approaches during the first iteration of the data evaluation process: first, between uMCD and boxplots directly, combining results with and without adjustment; then, between the adjusted and unadjusted results within each univariate method. For each set of results, the total number of outliers/errors by each approach is reported (uMCD vs. Boxplots; adjusted vs. unadjusted), as well as the number that overlapped between the two approaches. (DOCX 13 kb) [file 12874_2019_737_MOESM2_ESM.docx]

**Additional file 2: Comparison of outlying participants and errors identified by the univariate outlier detection approaches during the first iteration of the data evaluation process: first, between uMCD and boxplots directly, combining results with and without adjustment; then, between the adjusted and unadjusted results within each univariate method. For each set of results, the total number of outliers/errors by each approach is reported (uMCD vs. Boxplots; adjusted vs. unadjusted), as well as the number that overlapped between the two approaches.**

|  | | **Neuropsychology n = 161; p = 53** | | | **Gait n = 148; p = 29** | | |
| --- | --- | --- | --- | --- | --- | --- | --- |
| **Summary** | | **uMCD & Box.** | **uMCD** | **Box.** | **uMCD & Box.** | **uMCD** | **Box.** |
| **(Adj. & Unadj. are combined)** | **Outlying Participants** | 87 | 133 | 87 | 33 | 42 | 33 |
|  | **Number of Errors** | 1 | 3 | 1 | 3 | 3 | 3 |
| **Individual Results** | | **Adj. & Unadj.** | **Adj.** | **Unadj.** | **Adj. & Unadj.** | **Adj.** | **Unadj.** |
| **uMCD** | **Outlying Participants** | 113 | 120 | 126 | 31 | 38 | 35 |
|  | **Number of Errors** | 2 | 2 | 3 | 2 | 2 | 3 |
| **Boxplots** | **Outlying Participants** | 71 | 80 | 78 | 22 | 29 | 26 |
|  | **Number of Errors** | 1 | 1 | 1 | 2 | 2 | 3 |
